# Cost and time-effective method for multi-scale measures of rugosity, fractal dimension, and vector dispersion from coral reef 3D models

**DOI:** 10.1371/journal.pone.0175341

**Published:** 2017-04-13

**Authors:** G. C. Young, S. Dey, A. D. Rogers, D. Exton

**Affiliations:** 1 Department of Zoology, University of Oxford, Oxford, United Kingdom; 2 Operation Wallacea, Wallace House, Lincolnshire, United Kingdom; 3 ThinkSee3D Ltd., Eynsham, United Kingdom; University of Campinas, BRAZIL

## Abstract

We present a method to construct and analyse 3D models of underwater scenes using a single cost-effective camera on a standard laptop with (a) free or low-cost software, (b) no computer programming ability, and (c) minimal man hours for both filming and analysis. This study focuses on four key structural complexity metrics: point-to-point distances, linear rugosity (*R*), fractal dimension (*D*), and vector dispersion (*1/k*). We present the first assessment of accuracy and precision of structure-from-motion (SfM) 3D models from an uncalibrated GoPro^™^ camera at a small scale (4 m^2^) and show that they can provide meaningful, ecologically relevant results. Models had root mean square errors of 1.48 cm in X-Y and 1.35 in Z, and accuracies of 86.8% (*R*), 99.6% (*D* at scales 30–60 cm), 93.6% (*D* at scales 1–5 cm), and 86.9 (*1/k*). Values of *R* were compared to *in-situ* chain-and-tape measurements, while values of *D* and *1/k* were compared with ground truths from 3D printed objects modelled underwater. All metrics varied less than 3% between independently rendered models. We thereby improve and rigorously validate a tool for ecologists to non-invasively quantify coral reef structural complexity with a variety of multi-scale metrics.

## Introduction

Using an array of metrics in studies spanning decades, ecologists have shown that structural complexity drives biodiversity [1–4]. This is especially true on tropical coral reefs, one of the planet’s most biodiverse and productive ecosystems [5], where metrics of structural complexity correlate strongly with indicators of reef health such as fish abundance, coral, and macroalgal cover [6–8]. Causes and effects are intertwined in these cases: living things create structure, and structure must pre-exist for those living things to find shelter from predators, scavenge, avoid turbulence, or perform other actions necessary for them to thrive [9–12]. To more precisely understand the nature of the correlations between structural complexity and ecological parameters, for example in a way that could inform the design of artificial reefs, marine ecologists require precise tools for assessing 3D structure of underwater habitats. It is important and timely to develop and employ such tools because our window-of-opportunity is closing for studying healthy reef ecosystems: reef complexity has been shown to be in significant decline, leading to ecosystem collapse [13–16].

Popular methods of assessing underwater structural complexity include chain-and-tape rugosity and Habitat Assessment Scores (HAS) [17]. The most common of these is chain-and-tape rugosity, whereby a chain of known length is laid along the contours of the seabed, and the ratio of its draped to undraped length gives a rugosity value [18, 19]. Alternatively, divers can visually score a range of structural variables using HAS [17]. Although both methods have revealed correlations, they result in a cursory understanding of complexity that is inadequate for addressing fine-scale ecological questions [20] or informing artificial reef designs [21] and, moreover, may be fundamentally misleading because of factors such as observer bias and dimensionality reduction [2, 22, 23]. Marine researchers have long called for a modern method of assessing structural complexity to address these concerns [6, 8, 24]. Such a method could be incorporated into monitoring programs to improve time and cost efficiency, accuracy, and detail [25, 26].

Three-dimensional (3D) computer models are a solution for assessing reef structural complexity. 3D models generated from images via structure-from-motion (SfM) algorithms have successfully been implemented to assess terrestrial complexity (*e.g.,* [27, 28]). A few papers have presented methodologies for creating coral reef 3D models from cost-effective photogrammetry [16, 29–33]. The approach is not novel, but here we take the method further by (1) increasing its accessibility to a wider audience by reducing hardware and software costs, and (2) expanding the metrics that can be used by non-programmers to assess structural complexity and the quality of their 3D models. Expanding metrics gives researchers additional tools to answer ecological questions about 3D surfaces–*e.g.,* *At what scales does the structure provide refuge spots for prey to hide? How does a surface trap particulate matter?* We also show how footage from a single uncalibrated GoPro camera can produce 4 m^2^ 3D models with fine resolution and precision (to 1.5 cm with variations less than 3%).

We chose the SfM-software PhotoScan Standard (Agisoft LLC; $179 commercial, $59 educational—Mac, Windows, Linux; 30 day free trial) to render models for its ease-of-use and efficiency compared to its competitors and open-source alternatives; see discussion in [34]. We chose 3D modelling software Rhinoceros 3D (“Rhino”; Robert McNeel & Associates; $695–995 commercial, $195 educational—Mac, Windows; 90 day free trial) to analyse models for its easy-of-use, robustness, customizability, and library of built-in functions. Other software options were less favourable for a variety of reasons, including cost, platform limitations, and availability of a software development kit allowing us to write analysis scripts suited to our ecological applications.

Several papers emphasize the importance of calibration prior to photogrammetry, especially for highly distorted lenses such as that on a GoPro camera [35–37]. To date other studies using underwater photogrammetry have calibrated their cameras either from image meta-data that PhotoScan reads automatically or manual processing [30, 38, 39]. We show, however, that PhotoScan’s built-in proprietary algorithm (which uses Brown’s distortion model and no meta-data from our images), plus setting the camera to a narrow field-of-view, is capable of rendering accurate models at scales 1.5 cm—2 m. Because it does not assume camera calibration, this method could be used on historical footage, where camera model or calibration may be unknown.

We present the method alongside robust quantification of four structural variables: point-to-point distances, linear rugosity (*R*), fractal dimension (*D*), and vector dispersion (*1/k*). Per 4 m^2^ of modelled reef area, our method requires three minutes of in water filming time (with a single GoPro camera; ≈ $300) and approximately two hours of processing time on a standard laptop (≥8 GB RAM, 600 MB free disk space). Model rendering is largely automated, with each model requiring only 10 minutes human-computer time. We provide assessments of accuracy and repeatability using ground truths from known objects, as well as a comparison with chain-and-tape *in-situ* measurements of *R*. Our framework is ready-to-go to for use by non-programmers, and could be extended to gather any other conceivable structural complexity metric by a user with intermediate Python programming ability.

## Materials and methods

### Underwater filming

All filming occurred on reefs 5 ± 2 m deep off the Caribbean island of Utila, Honduras (16.0950^°^ N, 86.9274^°^ W) under a research permit from the Instituto de Conservación Forestal (#ICF-DE-MP-080-2016). We used GoPro cameras (Hero 3, 3+ or 4) in GoPro flat port underwater housings because they were readily available and widely used within contemporary reef monitoring efforts and recreational dive communities, although any similar camera should produce similar results. Cameras were in video mode, with all default settings except: resolution 1080p (for consistency across cameras), field-of-view narrow (to minimize distortion caused by the fish eye lens), sharpness medium (to minimize prominence of particulate matter), capture rate 24-30 frames per second, and white balance 6500K (for consistency and to suppress blue hues). Only ambient light illuminated scenes.

**Fig 1:**
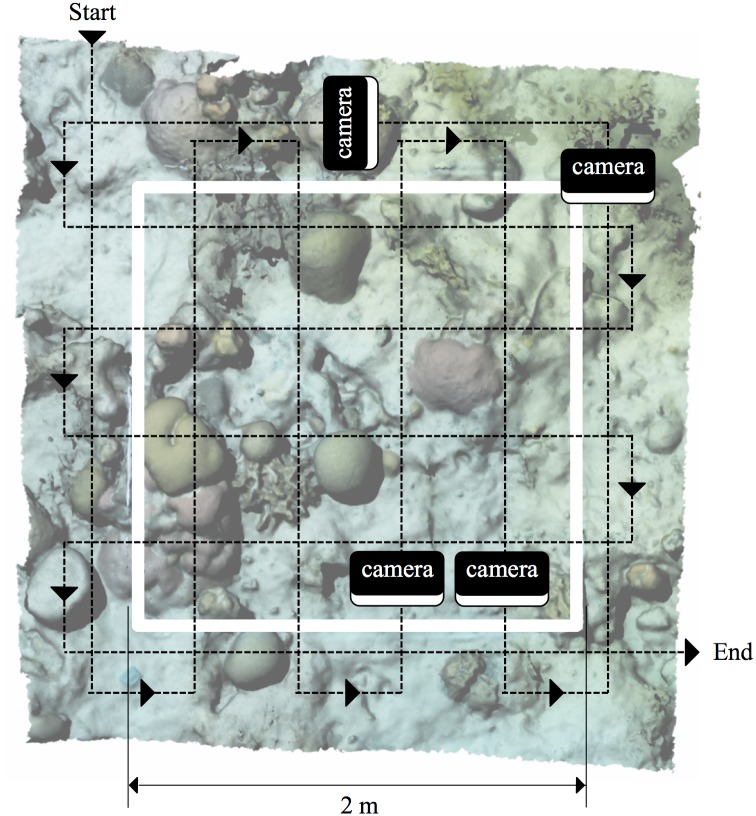
Method for filming 2 x 2 m underwater quadrat. A diver followed a lawnmower pattern (dotted line) over the quadrat, making 5–6 passes over each 2 m span of the quadrat and keeping the camera’s height and orientation consistent.

A SCUBA diver filmed over a 2 x 2 m quadrat following a lawnmower pattern (Fig 1). The camera remained a constant height 0.5–1.0 m above the scene’s highest point. It was aimed straight down at the substratum, the lens moving in one plane rather than following the contours of the scene. Underwater visibility needed only to be clear in the 0.5–1.0 m vertical distance between the camera and the reef, so even sites with relatively low visibility could be rendered. The orientation of the camera did not change between adjacent swim passes (Fig 1), meaning the diver either back-finned on an adjacent pass or held the camera still as he rotated his body.

### 3D model generation

We rendered models in PhotoScan following the standard process well described in the PhotoScan user manual and by other papers in the field (*e.g.,* [37]). Raw video footage was converted into sequences of still images using the free software FFmpeg (www.ffmpeg.org). Sequential images should contain 60–80% overlap, which in practice meant extracting at 3 frames per second. Approximately 300-600 images captured one 2 x 2 m quadrat.

Images loaded into PhotoScan were rendered into a 3D model following the standard process of (1) aligning photos, (2) building dense point cloud, (3) building mesh, and (4) building texture. All processes were set to medium quality with default settings, except meshes’ maximum face counts were set to 3,000,000 (to increase models’ fine-scale resolution). PhotoScan performs camera calibration automatically using Brown’s distortion model with assumed focal information. Photo alignment was successful even though we did not supply calibration information nor did the photos have EXIF data. Clarity of the model was then visually assessed. Any models in which the quadrat was not clear enough to be used as a scale bar would be rejected. However, no models rendered as part of this study needed to be rejected.

A rendered model was then exported as a wavefront (.OBJ) file and imported into Rhino for further analysis. Firstly, a model was scaled by setting a quadrat’s corner-to-corner length to 2 m using the Rhino “Scale” command. Secondly, the model was oriented using the “Rotate” command. For simplicity, we placed all quadrats flat underwater (*i.e.,*parallel to the ocean surface) and therefore rotated all models such that a quadrat corner rested squarely on the positive X and Y-axes. If a quadrat was placed at an angle underwater, however, divers could record the slope of the quadrat (*e.g.,* by tying a float indicating vertical or by recording the depths of the highest and lowest corners) and then rotate their 3D model accordingly.

### Assessment metrics

We analysed our 3D models in Rhino using four metrics: point-to-point distances, rugosity (*R*), fractal dimension (*D*), and vector dispersion (*1/k*). In addition, we assessed the precision of the models by repeatedly filming several scenes and quantifying variance between independent renderings. Students previously unfamiliar with the software involved learned to independently render and analyse models after a three-hour tutorial, so this method is suitable for rapid uptake.

#### Point-to-Point distances

**Fig 2:**
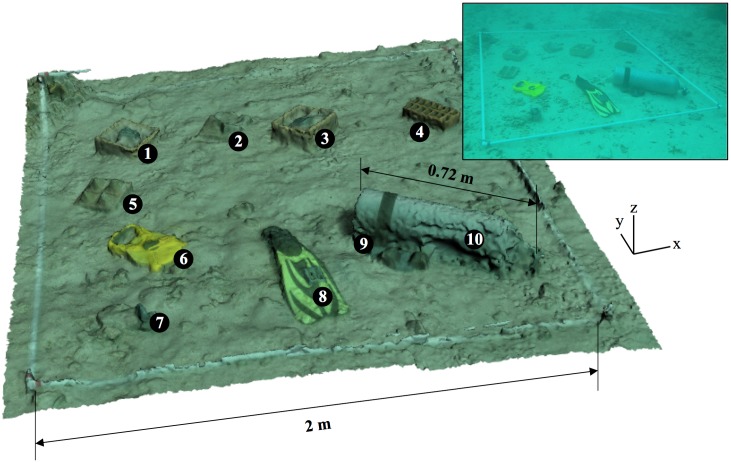
Objects of known dimensions 3D modelled inside 2 x 2 m quadrat. Two example dimensions, the quadrat length and the length of a standard SCUBA cylinder, are shown. Inset is a photo of the in-water scene. Objects of muted colors roughly matching the tones of the surrounding reef were chosen, representing a range of sizes and shapes. The 10 objects were: (1) pyramid-shaped mould, (2) pyramid-shaped tile, (3) natural-shaped mould, (4) brick, (5) pyramid-shaped tile, (6) transect tape, (7) dive weight (8) dive fin, (9) weight belt, and (10) SCUBA cylinder.

To demonstrate the proportional accuracy of our 3D models, we rendered an underwater scene containing ten man-made objects and compared objects’ known dimensions (ground truths) to their dimensions in the model. Objects of muted colors roughly matching the tones of the surrounding reef were chosen, representing a range of sizes and shapes (Fig 2). Known dimensions ranged from 0.8–65.0 cm in the X-Y plane and 2.0–18.0 cm in Z. No key dimensions were taken under an overhang, as an overhang will not render well using our filming method because we only move the camera in the X-Y plane, an issue further discussed below.

The accuracy of a measurement was computed with Eq 1:

$$\begin{array}{c} Accuracy=1-\frac{\left|UW\;3DM-Ground\;Truth\right|}{Ground\;Truth}\%, \end{array}$$ (1)

where *UW 3DM* is the dimension measured on the underwater 3D model, and *Ground Truth* is the known dimension.

#### Rugosity

We chose rugosity as a complexity metric because it is standard in traditional coral reef research—so standard that the most recent (as of March 2017) meta-analysis of structural complexity on reefs was only able to compare rugosities because of the “limited scale and replication” of studies employing alternative methods [8]. Rugosity is typically measured using the chain-and-tape method and quantified as the length the chain reaches as it falls over topography divided by the total length of the chain [18, 19]. It is not to be confused with “surface rugosity,” a term describing the ratio of 3D surface area to projected planar area, or with “roughness,” a term describing qualitative features or referencing the Hausdorff dimension.

To measure linear rugosity on a 3D model in Rhino, we first created a curve that followed the topography of the model. The curve was created with the Rhino built-in command “MeshIntersect,” which provides a cross-sectioning tool that allows the user to select a slice of user-determined linear length of the model by intersecting a mesh plane with the 3D reef mesh. In practice this can be performed between any two coordinates on the model. Here, in order to compare our 3D model-derived results with *in-situ* chain-and-tape measurements, curves were selectively positioned to match their in-water counterparts. We then ran a custom Rhino Python script (github.com/gracecalvertyoung/Rhino-Python-3D-Coral-Reefs/tree/master/Rugosity) using the “RunPythonScript” command. The script asked the user to select surface contours, and then it laid virtual chains along each contour and returned rugosities. Rugosity (*R*) equalled the distance that a virtual chain fell along the curve (R_*N*_) divided by the total length of the chain (R_*D*_). The virtual chain comprised of linear segments each the length of a chain link (2 cm was used), which the script created from the input contour with the build-in Rhino function “rs.DivideCurveEquidistant.”

**Fig 3:**
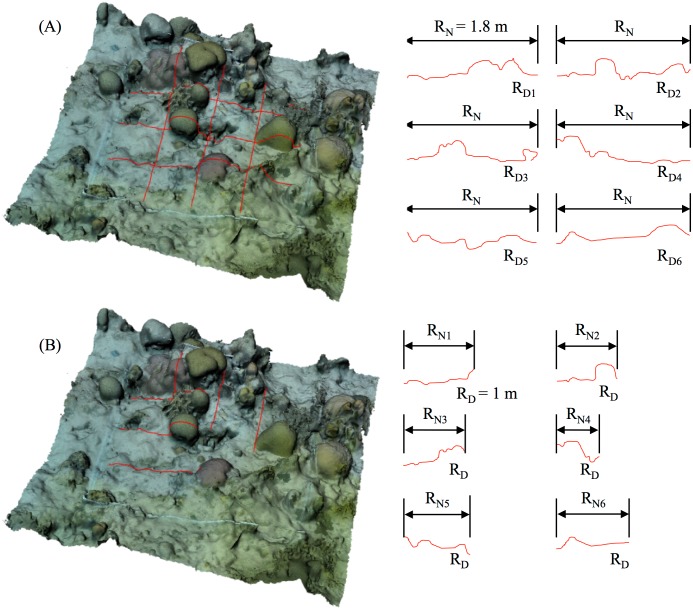
Methods for quantifying linar rugosity on 3D model. Six virtual chains with link length 2 cm were laid in a grid pattern over 3D modelled quadrats. A: The extendible-chain method determines how long a chain would need to be to cover the input curve. *R*_*N*_ is the draped length of the chain. *R*_*D*_*n*__ is the undraped length of chain *n*. B: The fixed-length chain method determines how far a chain of a set length (1 m was used) would reach over the curve; this method more closely resembles traditional chain-and-tape measurements, although it can miss details because of the chain’s limited length. *R*_*N*_*n*__ is the draped length of chain *n*. *R*_*D*_*n*__ is the undraped length of the chain.

The virtual chain was laid via either (1) the extendible-chain method (Fig 3A) or (2) the fixed-length chain method (Fig 3B). The extendible chain method determined how long a chain would need to be to cover the input curve, while the fixed-length chain method determined how far a chain of a set length (1 m was used) would fall along the input curve. This second method more closely resembled traditional chain-and-tape measurements, although it is a less accurate estimate of the overall reef complexity because of the chain’s limited length.

Results from both methods were compared against *in-situ* chain-and-tape measurements. For the purposes of this comparison, 3D model-derived rugosity was the average of three adjacent virtual chains spaced 4 cm apart to account for an *in-situ* chain not laying perfectly straight.

#### Fractal dimension

We choose fractal dimension (*D*) as a complexity metric because it is a sophisticated and accurate means of assessing surface complexity that has been shown to be well suited to describing coral reefs [40–43]. Developed by [44], *D* is between 2 and 3 for a surface, with a greater number indicating greater complexity. It allows structural complexity to be explored within set size categories; *e.g.,* researchers can define a size category based on a particular species of interest and its unique habitat requirements, or calculate complexity for multiple categories in a particular reef area [45, 46].

Contemporary studies in the field of pattern recognition (machine learning and/or computer vision) have presented alternatives to, or improvements on, traditional fractal dimension (*e.g.,* [47–49]), as further discussed under Future Study. However, these approaches are better suited to image analysis programs (*e.g.* MATLAB, as [50] uses) than within Rhino. Going from Rhino to an image analysis program for our use-case would introduce additional steps and software into the method. Therefore, in order to maintain a simplified and streamlined method while still providing useful ecological metrics, we choose to calculate *D* at multiple scales in Rhino.

There are several methods for calculating *D*, and different methods will yield different results [45, 50, 51]. While no method is definitively superior to all, [52, 53] suggest an area-based method is appropriate for calculating *D* of surfaces. We therefore implemented an area-based method, following [52], who estimated *D* of rock surfaces (an application similar to ours).

**Fig 4:**
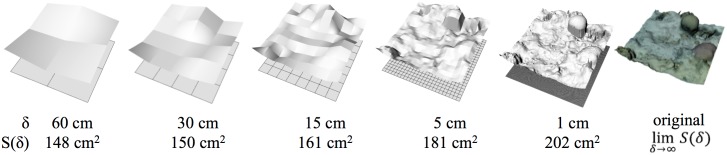
Fractal Dimension (*D*). *D* describes the relationship between a model’s resolution, or minimum pixel size, (*δ*) and its surface area S(*δ*). Above, the same patch of coral reef is rendered at five resolutions. The grid below each rendering is composed of squares, each of width *δ*, that are projected onto the original surface. Surface area always increases with finer resolution. *D* is 2—the slope logS(*δ*)/log(*δ*).

Following [52], *D* indicates how surface area changes with resolutions. It is the slope of a model’s resolution (*δ*) versus surface area (S(*δ*)) on logarithmic scales (Fig 4). We chose our resolutions, *δ* = 0.01, 0.05, 0.15, 0.3, 0.6, and 1.2 m, based on the refuge size categories of [17], who found holes of those size categories to be key factors influencing fish species abundance on coral reefs.

To measure *D* on a 3D model in Rhino, we ran a custom Rhino Python script (https://github.com/gracecalvertyoung/Rhino-Python-3D-Coral-Reefs/tree/master/Fractal_Dimension) using the “RunPythonScript” command. The script first re-rendered the model at resolution *δ* by projecting a grid of points spaced at *δ* onto the model, akin to dropping a blanket of points onto the model. The script then connected adjacent points to form a new, virtual quilt-like surface. The area of that surface was then plotted against *δ* on logarithmic scales and the slopes between points, or *D*, determined. For resolutions 0.05-0.01 m, D_0.05–0.01_ is (log(S(*δ* = 0.01)) − log(S(*δ* = 0.05)) / (log(0.01) − log(0.05)), and so on.

We compared 3D model-derived measurements of *D* to ground truths to gauge the accuracy and resolution of our 3D models in terms of *D*. The ground truths were three different theoretical structures that were 3D printed. The shapes placed underwater, hereafter refered to as the printed structures, matched the shapes of the 3D prints but were concrete, cast in moulds created from the 3D printed shapes. The accuracy of a measurement was calculated as a percentage (Eq 2).

$$\begin{array}{c} Accuracy_{D}=1-\frac{\left|UW\;3DM-Ground\;Truth\right|}{Ground\;Truth-1}\%, \end{array}$$ (2)

where *UW 3DM* is the value of *D* derived from the underwater 3D model and *Ground Truth* the value of *D* derived from the ground truth. Unlike Eq 1, one is subtracted from *Ground Truth* in the denominator because fractal dimension can only vary between 2 and 3 for a surface.

#### Vector dispersion

Vector dispersion (*1/k*) was determined as an appropriate metric for measuring benthic structural complexity by [54]. It measures the uniformity in angles of a surface. Mathematically, it estimates vector variance for all normal vectors of individual planar surfaces. It is a value between 0 and 1, where 0 indicates a flat plane and a number closer to 1 indicates a more complex surface. Like *R* and *D*, *1/k* must be calculated for a specified resolution; we choose 1 cm following [54]. In basic terms, a surface with a high value of *1/k* at 1 cm resolution would trap particulate matter more easily, be less easy to roll a ball of diameter 1 cm over (or clumps of sediment), and reflect light more variedly than a surface with a lower value of *1/k*.

**Fig 5:**
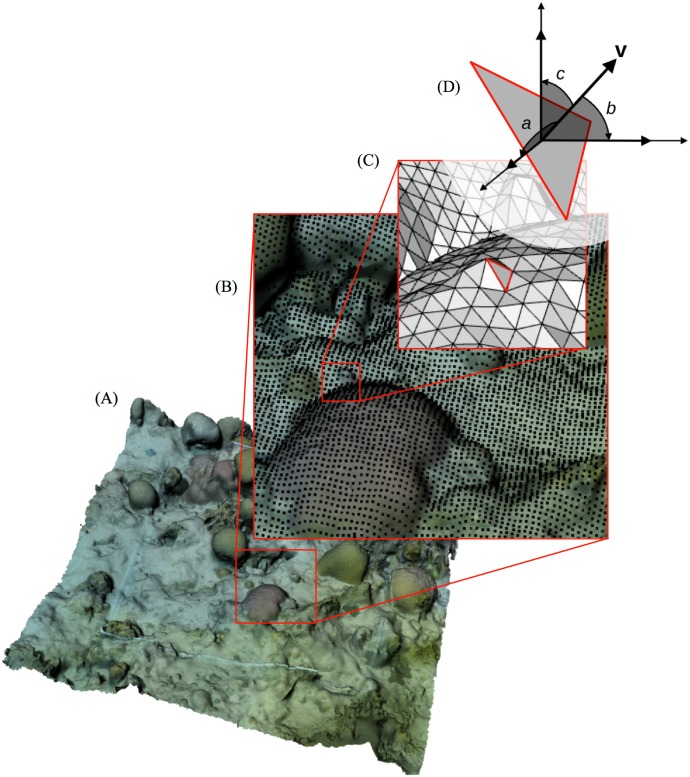
Process for computing vector dispersion (*1/k*). A: The user positions the scaled 3D model such that the quadrat lays flat along the X-Y plane or, if the quadrat was tilted underwater, tilted at the appropriate angle. The script then performs steps B-D. B: Project grid of points spaced 1 cm apart (as in [54]) onto the model such that each point falls on the highest point of the model. C: Connect adjacent points with triangles, creating *i* triangles. D: Compute the directional cosines of each triangle’s normal vector (*cos*_*x*_, *cos*_*y*_, and *cos*_*z*_ labelled a, b, and c in the inset), and combine them as in Eq 3 for *1/k*. Diagram D modified from material available through Creative Commons License.

To measure *1/k* in Rhino, we ran a custom Rhino Python script using the “RunPythonScript” command (https://github.com/gracecalvertyoung/Rhino-Python-3D-Coral-Reefs/tree/master/Vector_Dispersion). Whereas [54] created the grid of points with a profile gauge over the physical surface, our script projected a grid of points, spaced 1 cm apart, onto the highest Z-points of the 3D modelled reef. The script then created triangles between adjacent points and computed the directional cosines of triangles’ normal vectors (Fig 5). It then computed *1/k* using Eqs 3 and 4.

$$R1=\sqrt{{\left(\sum_{1}^{i}cos_{x}\right)}^{2}+{\left(\sum_{1}^{i}cos_{y}\right)}^{2}+{\left(\sum_{1}^{i}cos_{z}\right)}^{2}}$$ (3)

$$1/k=\frac{i-R1}{i-1},$$ (4)

where *i* is the number of triangles created between surface points and *cos*_*x*_ is the directional cosine of a triangle’s normal vector in the X direction, *cos*_*y*_ in the Y direction, and so on.

We compared *1/k* measurements from an underwater 3D model to ground truths (the same three printed structures used to validate measurements of *D*) in order to gauge the accuracy and resolution of our 3D models in terms of *1/k*.

#### Precision

We independently modelled eight 2 x 2 m quadrants three times each to evaluate the repeatability and consistency of our method in terms of the above metrics. *R* was computed using the extendible-chain method as the average of six virtual chains laid over the quadrat (as in Fig 3). *D* was computed for the resolutions *δ* = 0.01, 0.05, 0.15, 0.3, 0.6, and 1.2 m. *1/k* was computed as the average over 1.6 m^2^) of the quadrat.

## Results and discussion

The method took 3 minutes in-water filming per 2 x 2 m quadrant, significantly less time than did placing quadrats, laying out transect tape, or other activities of the dives. It was important that the diver keep the camera orientation consistent on adjacent swim passes (Fig 1) because if the diver instead rotated the camera with his body, the footage was too blurry or disparate for the SfM algorithm to render the model. We found that filming the perpendicular set of swim-overs (Fig 1) was necessary for consistently successful photo alignment, even though this step was not required by other studies that use SfM with diver-held monocular footage (*e.g.,* [37]). Our added step could be necessary because of our absence of calibration data, the small-scale of the quadrat, or non-manual intervention during photo alignment compared to other studies. The minimal time committed to this step (≈ 1.5 min dive time) made it worthwhile in ensuring model quality.

### Assessment metrics

#### Point-to-Point distances

**Fig 6:**
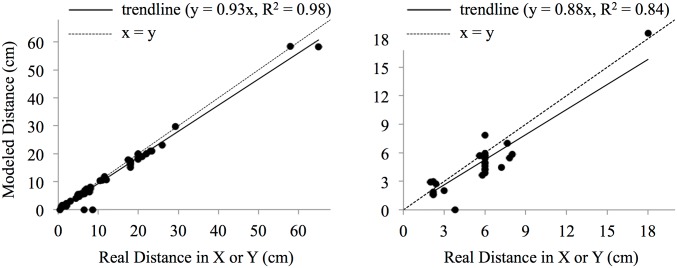
Accuracy of 3D model in terms of point-to-point distances. The root mean square errors (RMSE) of our models were 1.48 cm in X-Y and 1.35 cm in Z, with models underestimating dimensions in both X-Y and Z.

Dimensions on the underwater 3D model matched strongly with their true dimensions in both the X–Y (n = 48, R2 = 0.99; p < 0.001; Wilcoxon matched pairs test) and Z planes (n = 25, R2 = 0.83; p < 0.01; Wilcoxon matched pairs test). The root mean square errors (RMSE) of our models were 1.48 cm in X-Y and 1.35 cm in Z. The slopes of the regression plots (Fig 6) indicate that models underestimated dimensions in both X-Y and Z.

Measurements had accuracies of 89 ± 12% (mean ± SD) and 78 ± 13% in X-Y and Z respectively. These results are on-par with those of [30], who found their centimetre-scale underwater models to underestimate surface area and volume by 18% and 8% respectively. The improved accuracies of [30] were to be expected because they modelled smooth, bright, multicoloured objects in a tank of water, which should render better than natural scenes in the ocean.

Our accuracies are lower than what is possible from *in-situ* underwater SfM 3D models: [31] report RMSE errors of 0.605 mm from close-range photogrammetry from calibrated consumer-grade stereo-cameras. Our lower accuracy was to be expected, as we did not calibrate cameras and used a considerably less time-consuming rendering process compared to other methods: *e.g.,* methods that include manually removing outlier points on 3D models, manually identifying ground control objects, and/or using PhotoScan’s high or ultra-high quality settings. [55] showed that models from calibrated GoPro footage can achieve RMSE errors of 0.40 mm; users requiring models accurate at scales finer than 1.5 cm should consider a method requiring higher hardware/software effort and cost or at minimum calibrate their cameras.

Our reduced accuracy in Z compared to X-Y was expected because the camera travelled only in the X-Y plane. Having divers film around objects to capture more Z-plane features may improve Z-plane accuracies as well as capture structures precluded by overhangs. We performed a few trials filming perpendicular to the surface terrain, or *around* objects, but it led to unwanted noise in the models or yielded unsuccessful photo alignment—complications likely caused by the extended background water column introducing moving particulates and not containing features for the SfM algorithm to align. A solution could be PhotoScan’s “mask” feature, but trade-offs with dive time and ease-of-use would need to be considered.

Overall the results indicate that measurements taken from 3D modelled reef in any direction can be treated with a high degree of confidence. This is further supported by the consistently accurate results obtained from the varying selection of shaped and sized objects, which gives reassurances when working with the highly variable structure of the natural world. It is worth noting that some objects rendered better than others. For example, looking closely at Fig 2, the surface of the SCUBA tank appears to have a texture less smooth than the real life object; this could be because it is somewhat shiny and therefore not ideal for photogrammetry. These texture discrepancies are better estimated by the metrics of rugosity, fractal dimension and vector dispersion rather than point-to-point distances, however, and so are discussed in more detail later. Importantly at this stage, it had no impact on the accuracy of point-to-point distance measurements, meaning our models are well suited to the collection of size data.

#### Rugosity

**Fig 7:**
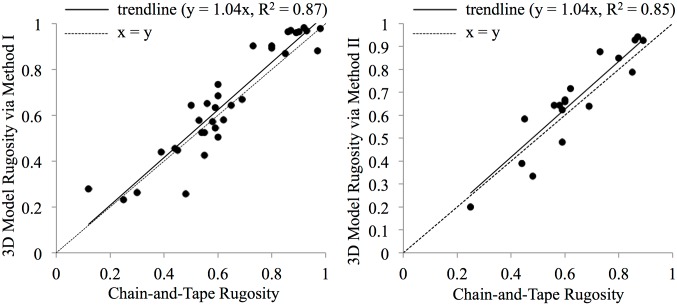
*In-situ* chain-and-tape measurements compared to those taken with a virtual chain on a 3D model. Method I is the extendible-chain method and Method II is the fixed-length chain method. The extendible chain method had an accuracy of 85.7 ± 22.8% and the fixed length chain method had an accuracy of 86.8 ± 7.8%.

Models’ rugosities matched strongly with traditional *in-situ* chain-and-tape measurements (Fig 7), both via the extendible-chain method (n = 34, R^2^ = 0.86; p < 0.001; one-sample t-test) and the fixed-length chain method (n = 18, R^2^ = 0.83; p < 0.001; one-sample t-test).

Reported as accuracies using Eq 1, the extendible chain method had an accuracy of 85.7 ± 22.8% and the fixed length chain method had an accuracy of 86.8 ± 7.8%. These accuracies are on par with the accuracies of 85.3 ± 0.6% [33] and 89% [56], the only other studies to compare linear rugosity from a SfM 3D model (albeit using different methods) to chain-and-tape measurements.

#### Fractal dimension

**Fig 8:**
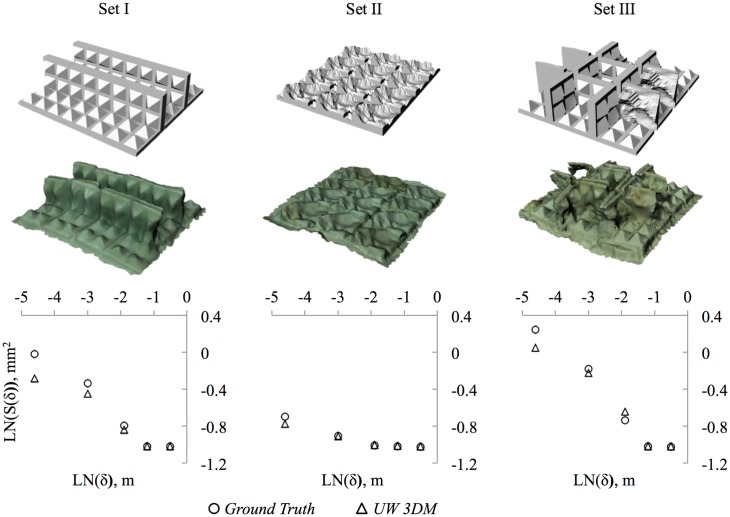
Fractal Dimension (*D*) of underwater 3D-printed objects at five spacial scales compared to ground truths. 3D printed structures were placed underwater and 3D modelled. Their surface areas were computed at five spatial scales (60, 30, 15, 5, and 1 cm) to compute *D*, which is the slope of model’s resolution versus model’s surface area on logarithmic scales. Surface areas at the 60 and 30 cm resolutions matched nearly perfectly between the ground-truth structures (top row) and the underwater 3D models (*UW 3DM*), while the 3D models slightly underestimated surface area at finer resolutions.

*D* values from underwater 3D models matched well with ground truths. The highest accuracy occurred for measurements at the largest measured resolution, 30–60 cm (99.67 ± 0.11%) and accuracy decreased only to 93.57 ± 2.13% at the finest resolution, 1–5 cm (Fig 8; Table 1). Reduced accuracy at the finer scale was understandable, as smaller details are logically more difficult to capture because of complications such as particulate matter in the water interfering with image resolution. *D* values were marginally underestimated at the 1–30 cm resolutions, which is visibly demonstrated by the excessively smooth appearance of modelled objects (Fig 8).

**Table 1:** Accuracies of Underwater 3D Models (*UW 3DM*) in terms of fractal Dimension (*D*) and vector dispersion (*1/k*). Accuracies computed using Eq 2 for *D* and Eq 1 for *1/k*. Sets I, II, and III are pictured in Fig 8.

Measurement	Accuracy (%)	Set	*Ground Truth*	*UW 3DM*
*D*_0.60−0.30_	99.67 ± 0.11	I	2.0032	2.0056
		II	2.0026	2.0071
		III	2.0003	2.0033
*D*_0.30−0.15_	95.26 ± 4.59	I	2.3257	2.2606
		II	2.0131	2.0138
		III	2.4096	2.54
*D*_0.15−0.05_	93.26 ± 2.01	I	2.4148	2.3516
		II	2.0952	2.0138
		III	2.5085	2.383
*D*_0.05−0.01_	93.57 ± 2.13	I	2.1975	2.1044
		II	2.131	2.086
		III	2.264	2.1687
*1/k*	86.94 ± 4.55	I	0.249	0.226
		II	0.194	0.171
		III	0.304	0.359

The higher accuracy of *D* compared to point-to-point distances, rugosity, and *1/k* indicates that models are well-suited to convey overall complexity, even though some features may not perfectly match their ground truths.

#### Vector dispersion

*1/k* matched well with ground truths, with an overall accuracy of 86.94 ± 4.55% (Table 1). There was no consistency in whether the models over- or underestimated *1/k*. This level of accuracy can be considered above satisfactory, and further validates that the underwater 3D models used here accurately represent the structural complexity of their study areas. While no other study to our knowledge has computed accuracies in terms of *D* or *1/k*, our accuracies are on the high-end of the wide range reported by other studies computing surface area and volume from photographic models, accuracies which range 1–17% and 2–9% for surface area and volume, respectively [38].

### Precision

The rendering of multiple models of the same reef area demonstrated the high repeatability of the method. Table 2 shows that coefficient of variation (CV) in measurements were all below 2.8% (for rugosity) and as low as 0.6% (for D between 1–5 cm). The slight variations could result from human influences such as inconsistency in filming technique, scaling along the quadrat, and placement of rugosity lines or point clouds over the model. While no other study to our knowledge has computed precision in terms of *D* or *1/k*, our results are on the low-end of the range of 1–10% reported by [38] for surface rugosity from photogrammetric models. Similar to our method, [38] modelled six scenes 7–10 times to derive their CVs.

**Table 2:** Precision of 3D models. Models showed low variation in terms of rugosity (*R*), vector dispersion (*1/k*) and fractal Dimension (*D*). Eight quadrats were each modelled three times. The coefficient of variation (CV) was the average standard deviation of measurements divided by the average measurement.

	*R*	*D*_1.20−0.60_	*D*_0.60−0.30_	*D*_0.30−0.15_	*D*_0.15−0.05_	*D*_0.05−0.01_	*1/k*
CV	2.8%	0.7%	1.3%	1.0%	2.8%	0.6%	1.9%

### Future study

To further refine the filming technique and model rendering process, it would be helpful to assess the accuracy of measurements along the Z plane for larger objects, as this study only measured up to 18 cm in Z. It would also be useful to explore how model quality is affected by water conditions, available lighting (quality/quantity), depth, and other environmental factors (*e.g.,* [57] look at sun and wind patterns to find the optimum daytime for filming).

Computing additional metrics of structural complexity could also assist with long-term reef monitoring strategies and benthic community assessments. Metrics such as surface area, volume, slope, and average height could be of interest, as could any of those reviewed by [2] or [58]. Slope in particular is not addressed in our study, as all our quadrats were placed flat (parallel to the ocean surface) for simplicity and consistency. To incorporate slope into 3D models, the models should be rotated to the appropriate angle prior to obtaining metrics, or rugosity could be decoupled from slope [56]. We attempted to tie a small fishing float to the corner of a quadrat to indicate its angle with respect to the surface, but, unsurprisingly, the float moved too much to render in the 3D model. On future studies, divers might record a quadrat’s angle by noting the depths of two corners of the quadrat and positioning the 3D model accordingly. Advanced users may also wish to implement other numerical approaches for estimating fractal dimension and/or metrics from the field of pattern recognition (machine learning and/or computer vision) such as lacunarity [47], color texture analysis based on fractal geometry [48], and/or local fractal dimension [49]. These state-of-the-art methods are presented for image analysis, but it would be possible to apply them on a coral reef 3D model by converting the 3D model into a “heat map” or 2D array of the quadrat. Once the heat map is generated, existing toolboxes in MATLAB (*e.g.,* as [50] uses) would likely be more suited to the calculations than Rhino-Python scripts. That said, a user would still need to initially process the model in Rhino to scale, rotate, and identify the quadrat area.

## Conclusion

While 3D modelling from underwater photogrammetry is a reasonably established method for representing and assessing coral reef structures, it remains largely reliant on sophisticated or costly hardware and/or software that can restrict accessibility to the wider research and conservation community. We present a cost-effective and automated technique that demonstrates how a single uncalibrated GoPro camera can produce accurate and precise models at small spatial scales (1.5 cm to 2 m), with variations in structural complexity between models below 3% and a high level of accuracy when compared to ground-truth measurements. We also provide useful tools for non-programmers to quantify reef 3D structures via a suite of ecologically-relevant metrics. By expanding beyond simple rugosity measurements to include fractal dimension (*D*) and vector dispersion (*1/k*), we provide researchers with a more thorough approach to exploring the quantity and quality of 3D complexity, including the ability to focus on complexity ranges that are ecologically relevant to target organisms.

## References

[1] Hiatt RW, Strasburg DW, Monographs SE, Jan N. Ecological Relationships of the Fish Fauna on Coral Reefs of the Marshall Islands. Ecological Monographs. 1960;30(1):65–127. 10.2307/1942181

[2] Mccormick MI. Comparison of Field Methods for Measuring Surface Topography and their Associations with a Tropical Reef Fish Assemblage. Marine Ecology Progress Series. 1994;112:87–96. 10.3354/meps112087

[3] Knudby A, LeDrew E. Measuring Structural Complexity on Coral Reefs. Proceedings of the American Acadamy of Underwater Sciences 26th Symposium. 2007; p. 181–188.

[4] Dustan P, Doherty O, Pardede S. Digital Reef Rugosity Estimates Coral Reef Habitat Complexity. PLoS ONE. 2013;8(2). 10.1371/journal.pone.0057386 23437380 PMC3578865

[5] Alvarez-Filip L, Dulvy NK, Côteé IM, Watkinson AR, Gill Ja. Coral Identity Underpins Architectural Complexity on Caribbean Reefs. Ecological Applications. 2011;21(6):2223–2231. 10.1890/10-1563.1 21939056

[6] Friedlander AM, Parrish JD. Habitat Characteristics Affecting Fish Assemblages on a Hawaiian Coral Reef. Journal of Experimental Marine Biology and Ecology. 1998;224(1):1–30. 10.1016/S0022-0981(97)00164-0

[7] Wilson MFJ, O’Connell B, Brown C, Guinan JC, Grehan AJ. Multiscale Terrain Analysis of Multibeam Bathymetry Data for Habitat Mapping on the Continental Slope. Marine Geodesy. 2007;30(1–2):3–35. 10.1080/01490410701295962

[8] Graham NAJ, Nash KL. The Importance of Structural Complexity in Coral Reef Ecosystems. Coral Reefs. 2013;32:315–326. 10.1007/s00338-012-0984-y

[9] Hixon MA, Beets JP. Predation, Prey Refuges, and the Structure of Coral-Reef Fish Assemblages. Ecological Monographs. 1993;63(1):77–101. 10.2307/2937124

[10] Carr MH, Hixon MA. Artificial Reefs: The Importance of Comparisons with Natural Reefs. Fisheries. 1997;22(4):28–33. 10.1577/1548-8446(1997)022<0028:ARTIOC>2.0.CO;2

[11] Hearn C, Atkinson M, Falter J. A physical derivation of nutrient-uptake rates in coral reefs: Effects of roughness and waves. Coral Reefs. 2001;20(4):347–356. 10.1007/s00338-001-0185-6

[12] Johansen JL, Bellwood DR, Fulton CJ. Coral reef fishes exploit flow refuges in high-flow habitats. Marine Ecology Progress Series. 2008;360:219–226. 10.3354/meps07482

[13] Alvarez-Filip L, Dulvy NK, Gill Ja, Cote IM, Watkinson aR. Flattening of Caribbean Coral Reefs: Region-Wide Declines in Architectural Complexity. Proceedings of the Royal Society B: Biological Sciences. 2009;276(1669):3019–3025. 10.1098/rspb.2009.0339 19515663 PMC2817220

[14] Ledlie MH, Graham NAJ, Bythell JC, Wilson SK, Jennings S, Polunin NVC, et al Phase shifts and the role of herbivory in the resilience of coral reefs Coral Reefs. 2007; p. 641–653. 10.1007/s00338-007-0230-1

[15] Newman SP, Meesters EH, Dryden CS, Williams SM, Sanchez C, Mumby PJ, et al Reef Flattening Effects on Total Richness and Species Responses in the Caribbean. Journal of Animal Ecology. 2015;84(6):1678–1689. 10.1111/1365-2656.12429 26344713

[16] Burns JHR, Delparte D, Kapono L, Belt M. Assessing the impact of acute disturbances on the structure and composition of a coral community using innovative 3D reconstruction techniques. Methods in Oceanography. 2016; p. 1–11.

[17] Gratwicke B, Speight MR. The Relationship Between Fish Species Richness, Abundance and Habitat Complexity in a Range of Shallow Tropical Marine Habitats. Journal of Fish Biology. 2005;66(3):650–667. 10.1111/j.0022-1112.2005.00629.x

[18] Risk MJ. Fish Diversity on a Coral Reef in The Virgin Islands. Atoll Research Bulletin. 1972;153:1–6. 10.5479/si.00775630.153.1

[19] Luckhurst E, Luckhurst K. Analysis of the Influence of Substrate Variables on Coral Reef Fish Communities. Marine Biology. 1978;323(49):317–323. 10.1007/BF00455026

[20] Harborne AR, Mumby PJ, Ferrari R. The effectiveness of different meso-scale rugosity metrics for predicting intra-habitat variation in coral-reef fish assemblages. Environmental Biology of Fishes. 2012;94(2):431–442. 10.1007/s10641-011-9956-2

[21] Perkol-Finkel S, Shashar N, Benayahu Y. Can artificial reefs mimic natural reef communities? The roles of structural features and age. Marine Environmental Research. 2006;61(2):121–135. 10.1016/j.marenvres.2005.08.001 16198411

[22] Goatley CHR, Bellwood DR. The Roles of Dimensionality, Canopies and Complexity in Ecosystem Monitoring. PLoS ONE. 2011;6(11). 10.1371/journal.pone.0027307 PMC3207849 22073311

[23] Kerry JT, Bellwood DR. The effect of coral morphology on shelter selection by coral reef fishes. Coral Reefs. 2012;31(2):415–424. 10.1007/s00338-011-0859-7

[24] Merks R, Hoekstra A, Kaandorp J, Sloot P. A Problem Solving Environment for Modelling Stony Coral Morphogenesis. Computational Science—ICCS 2003. 2003;2657:639–648. 10.1007/3-540-44860-8_66

[25] Wedding LM, Friedlander AM, McGranaghan M, Yost RS, Monaco ME. Using Bathymetric Lidar to Define Nearshore Benthic Habitat Complexity: Implications for Management of Reef Fish Assemblages in Hawaii. Remote Sensing of Environment. 2008;112(11):4159–4165. 10.1016/j.rse.2008.01.025

[26] Mumby P, Flower J, Chollett I, Box S, Bozec Y. Towards reef resilience and sustainable livelihoods: A handbook for Caribbean Coral Reef Managers. Exeter, Devon, UK: University of Exeter; 2014.

[27] Westoby MJ, Brasington J, Glasser NF, Hambrey MJ, Reynolds JM. ‘Structure-from-Motion’ photogrammetry: A low-cost, effective tool for geoscience applications. Geomorphology. 2012;179:300–314. 10.1016/j.geomorph.2012.08.021

[28] Javernick L, Brasington J, Caruso B. Modeling the topography of shallow braided rivers using Structure-from-Motion photogrammetry. Geomorphology. 2014;213:166–182. 10.1016/j.geomorph.2014.01.006

[29] Hu H, Ferrari R, Mckinnon D, Roff Ga, Smith R, Mumby PJ, et al Measuring reef complexity and rugosity from monocular video bathymetric reconstruction. International Coral Reef Symposium. 2012;12(July):9–13.

[30] Lavy A, Eyal G, Neal B, Keren R, Loya Y, Ilan M. A quick, easy and non-intrusive method for underwater volume and surface area evaluation of benthic organisms by 3D computer modelling. Methods in Ecology and Evolution. 2015; p. n/a–n/a. 10.1111/2041-210X.12331

[31] Leon JX, Roelfsema CM, Saunders MI, Phinn SR. Measuring Coral reef Rerrain Roughness using ‘Structure-from-Motion’ Close-Range Photogrammetry. Geomorphology. 2015;242:21–28. 10.1016/j.geomorph.2015.01.030

[32] Storlazzi CD, Dartnell P, Hatcher GA, Gibbs AE. End of the chain? Rugosity and fine-scale bathymetry from existing underwater digital imagery using structure-from-motion (SfM) technology Coral Reefs. 2016;. 10.1007/s00338-016-1462-8

[33] Ferrari R, McKinnon D, He H, Smith RN, Corke P, Gonzalez-Rivero M, et al Quantifying multiscale habitat structural complexity: A cost-effective framework for underwater 3D modelling. Remote Sensing. 2016;8(2). 10.3390/rs8020113

[34] Dandois JP, Ellis EC. High spatial resolution three-dimensional mapping of vegetation spectral dynamics using computer vision. Remote Sensing of Environment. 2013;136:259–276. 10.1016/j.rse.2013.04.005

[35] Balletti C, Guerra F, Tsioukas V, Vernier P. Calibration of action cameras for photogrammetric purposes. Sensors. 2014;14(9):17471–17490. 10.3390/s140917471 25237898 PMC4208234

[36] Helmholz P, Long J, Munsie T, Belton D. Accuracy assessment of go pro hero 3 (Black) camera in underwater environment. International Archives of the Photogrammetry, Remote Sensing and Spatial Information Sciences—ISPRS Archives. 2016;41(July):477–483. 10.5194/isprs-archives-XLI-B5-477-2016

[37] Burns J, Delparte D, Gates R, Takabayashi M. Integrating Structure-from-Motion Photogrammetry with Geospatial Software as a Novel Technique for Quantifying 3D Ecological Characteristics of Coral Reefs. PeerJ. 2015; 10.7717/peerj.1077 26207190 PMC4511817

[38] Figueira W, Ferrari R, Weatherby E, Porter A, Hawes S, Byrne M. Accuracy and Precision of Habitat Structural Complexity Metrics Derived from Underwater Photogrammetry. Remote Sensing. 2015;7(12):16883–16900. 10.3390/rs71215859

[39] Gutierrez-Heredia L, Benzoni F, Murphy E, Reynaud EG. End to End Digitisation and Analysis of Three-Dimensional Coral Models, from Communities to Corallites. PLoS ONE. 2016;11(2). 10.1371/journal.pone.0149641 26901845 PMC4763093

[40] Bradbury RH, Reichelt E. Fractal Dimension of a Coral Reef. Marine Ecology Progress Series. 1983;10:169–171. 10.3354/meps010169

[41] Mark D. Fractal Dimension of a Coral Reef at Ecological Scales: A Discussion. Marine Ecology Progress Series. 1984;14:293–294. 10.3354/meps014293

[42] Herzfeld UC, Overbeck C. Analysis and simulation of scale-dependent fractal surfaces with application to seafloor morphology. Computers and Geosciences. 1999;25:979–1007. 10.1016/S0098-3004(99)00062-X

[43] Martin-Garin B, Lathuilière B, Verrecchia EP, Geister J. Use of Fractal Dimensions to Quantify Coral Shape. Coral Reefs. 2007;26(3):541–550. 10.1007/s00338-007-0256-4

[44] Mandelbrot BB. The Fractal Geometry of Nature. New York: W.H. Freeman; 1982.

[45] Kostylev VE, Erlandsson J, Mak YM, Williams GA. The relative importance of habitat complexity and surface area in assessing biodiversity: Fractal application on rocky shores. Ecological Complexity. 2005;2(3):272–286. 10.1016/j.ecocom.2005.04.002

[46] Zawada DG, Brock JC. A Multiscale Analysis of Coral Reef Topographic Complexity Using Lidar-Derived Bathymetry. Journal of Coastal Research. 2009;10053:6–15. 10.2112/SI53-002.1

[47] Hsui CY, Wang CC. Synergy between fractal dimension and lacunarity index in design of artificial habitat for alternative SCUBA diving site. Ecological Engineering. 2013;53:6–14. 10.1016/j.ecoleng.2013.01.014

[48] Casanova D, Florindo JB, Falvo M, Bruno OM. Texture analysis using fractal descriptors estimated by the mutual interference of color channels. Information Sciences. 2016;346–347:58–72. 10.1016/j.ins.2016.01.077

[49] Novianto S, Suzuki Y, Maeda J. Near optimum estimation of local fractal dimension for image segmentation. Pattern Recognition Letters. 2003;24(1–3):365–374. 10.1016/S0167-8655(02)00261-1

[50] Gneiting T, Ševčíková H, Percival DB. Estimators of Fractal Dimension: Assessing the Roughness of Time Series and Spatial Data. Statistical Science. 2012;27(2):254–282. 10.1214/11-STS370

[51] Klinkenberg B, Goodchild MF. The fractal properties of topography: A comparison of methods. Earth Surface Processes and Landforms. 1992;17(3):217–234. 10.1002/esp.3290170303

[52] Zhou HW, Xie H. Direct Estimation of the Fractal Dimensions of a Fracture Surface of Rock. Surface Review and Letters. 2003;10(05):751–762. 10.1142/S0218625X03005591

[53] Zhou G, Lam NSN. A comparison of fractal dimension estimators based on multiple surface generation algorithms. Computers and Geosciences. 2005;31(10):1260–1269. 10.1016/j.cageo.2005.03.016

[54] Carleton JH, Sammarco PW. Effects of Substratum Irregularity on Success of Coral Settlement: Quantification by Comparative Geomorphological Techniques. Bulletin of Marine Science. 1987;40(1):85–98.

[55] Guo T, Capra A, Troyer M, Gruen A, Brooks AJ, Hench JL, et al Accuracy Assessment of Underwater Photogrammetric Three Dimensional Modelling for Coral Reefs. ISPRS—International Archives of the Photogrammetry, Remote Sensing and Spatial Information Sciences. 2016;XLI-B5(June):821–828. 10.5194/isprsarchives-XLI-B5-821-2016

[56] Friedman A, Pizarro O, Williams SB, Johnson-Roberson M. Multi-Scale Measures of Rugosity, Slope and Aspect from Benthic Stereo Image Reconstructions. PLoS ONE. 2012;7(12). 10.1371/journal.pone.0050440 23251370 PMC3520945

[57] Casella E, Collin A, Harris D, Ferse S, Bejarano S, Parravicini V, et al Mapping coral reefs using consumer-grade drones and structure from motion photogrammetry techniques Coral Reefs. 2016; p. 1–7.

[58] Pittman SJ, Brown KA. Multi-Scale Approach for Predicting Fish Species Distributions across Coral Reef Seascapes. PLoS ONE. 2011;6(5):e20583 10.1371/journal.pone.0020583 21637787 PMC3102744

