# Correction: Cost and time-effective method for multi-scale measures of rugosity, fractal dimension, and vector dispersion from coral reef 3D models

**DOI:** 10.1371/journal.pone.0201847

**Published:** 2018-07-31

**Authors:** G. C. Young, S. Dey, A. D. Rogers, D. Exton

Fig 6 is incorrect. The authors have provided a corrected version here.

**Fig 6:**
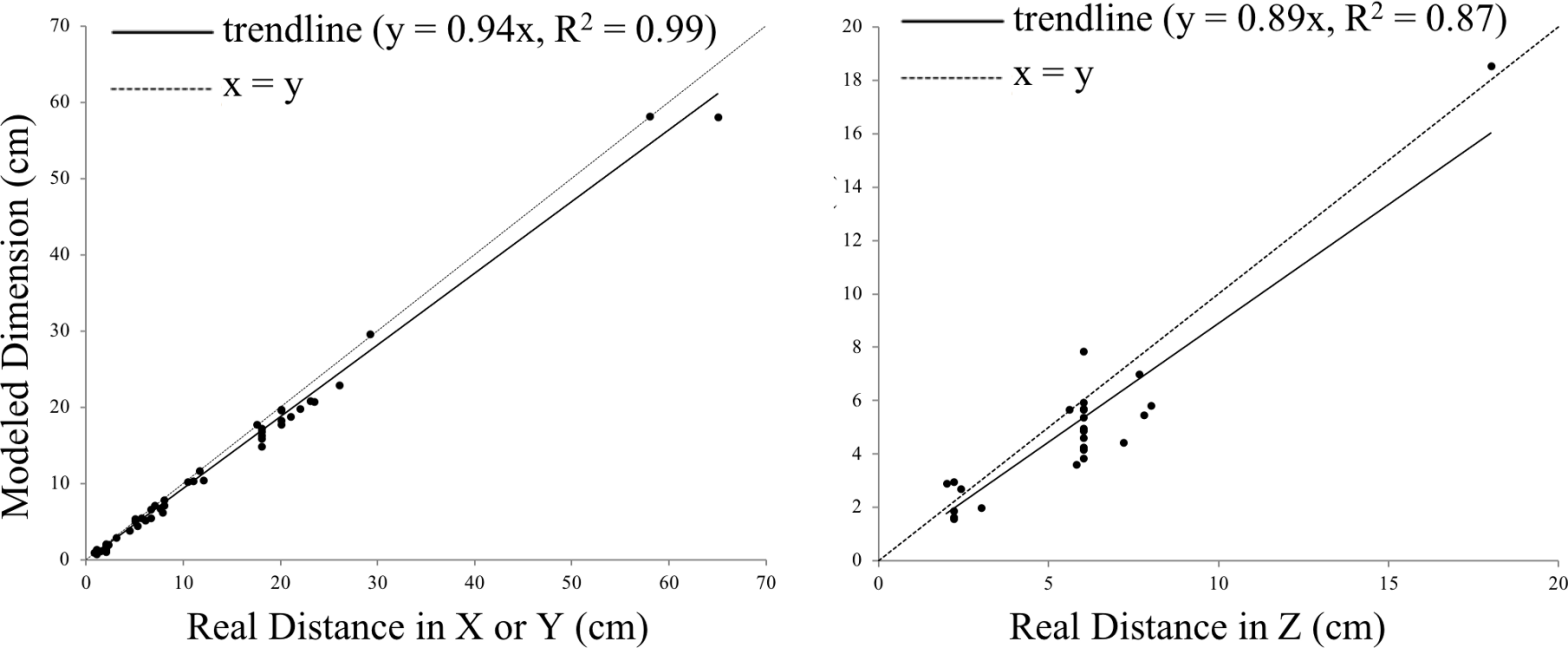
Accuracy of 3D model in terms of point-to-point distances. The root mean square errors (RMSE) of our models were 1.48 cm in X-Y and 1.35 cm in Z, with models underestimating dimensions in both X-Y and Z.
